# Supplementary material for: Genomic GC bias correction improves species abundance estimation from metagenomic data
Source: Nat Commun. 2025 Nov 26;16:10523. doi: 10.1038/s41467-025-65530-4 (PMC12658245; doi:10.1038/s41467-025-65530-4)
Supplement: Supplementary file 1 — Supplementary Information [file 41467_2025_65530_MOESM1_ESM.pdf]

Genomic GC bias correction improves species abundance estimation from metagenomic data

Supplementary Information

Laurenz Holcik, Arndt von Haseseler, Florian G. Pflug

List of Tables

S1 Runtimes for Metagenomic Analysis Tools 2

List of Figures

S1 GC-bias depends on the sequencing platform 2
S2 Sequencing efficiency of *Fusobacterium* in single-species libraries . . . 2
S3 Individual GC-dependent sequencing efficiencies of Fig. 4 3
S4 Clustering of the studies of Fig. 4 3
S5 Difference in alpha diversity across studies 4
S6 Inferred taxon-specific efficiencies 4
S7 False positive and negative taxa detection 5
S8 Average abundance and read fraction removed by FP detection . . . 6

Analysis Tool	Runtime	Cores Used
GuaCAMOLE	4m 54s	1
Sylph	1m 10s	1
mOTUS	6m 25s	10
Kraken2	3m 27s	10
MetaPhlAn4	6m 11s	10
Bracken	0.35s	1

Table S1 Runtimes for Metagenomic Analysis Tools on a Sample of 9.3 Mio read pairs. Note that Bracken as well as GuaCAMOLE require Kraken2 to be run beforehand.

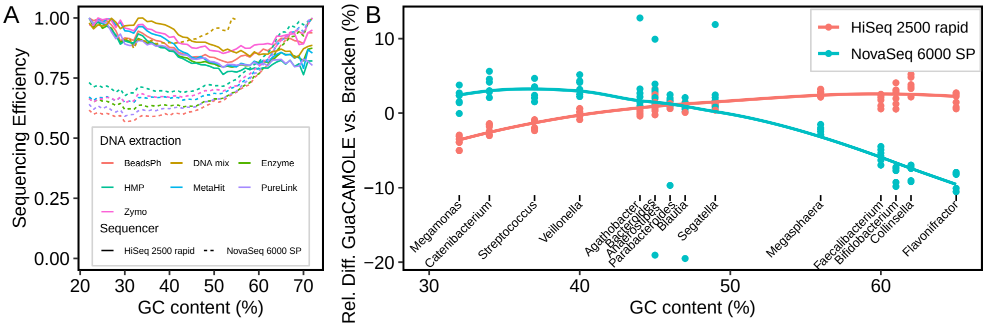

Fig. S1 GC-bias depends on the sequencing platform. Mori *et al.* sequenced a mock community comprising 18 bacterial species with a wide range of GC contents using 7 different DNA extraction methods and two different sequencing platforms [1]. **(A)** GC-dependent sequencing efficiencies for the tested protocols and sequencing platforms. **(B)** Relative difference between the abundances reported by Bracken and GuaCAMOLE for genera with different GC content.

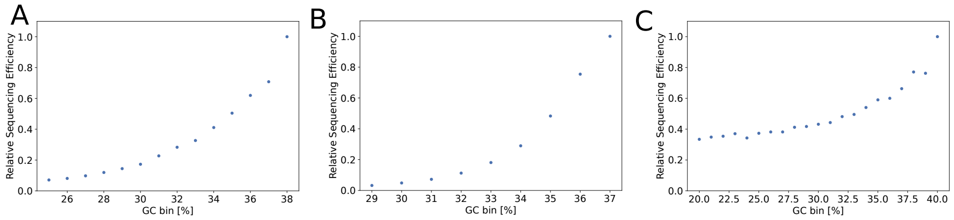

Fig. S2 Sequencing efficiency of *Fusobacterium* in single-species libraries. Sequencing efficiencies predicted by GuaCAMOLE for single-species libraries of Browne et al. [2] containing only *Fusobacterium sp. C1* (accessions SRR8257183, SRR8257184 and SRR8257185) sequenced on different platforms. RefSeq does not contain a genome for *Fusobacterium sp. C1*, and consequently most reads were assigned by Kraken2 to *Fusobacterium hominis*. The efficiencies estimated by GuaCAMOLE agree with those reported by Browne et al. **(A)** Illumina NextSeq. **(B)** Illumina MiSeq. **(C)** Illumina HiSeq.

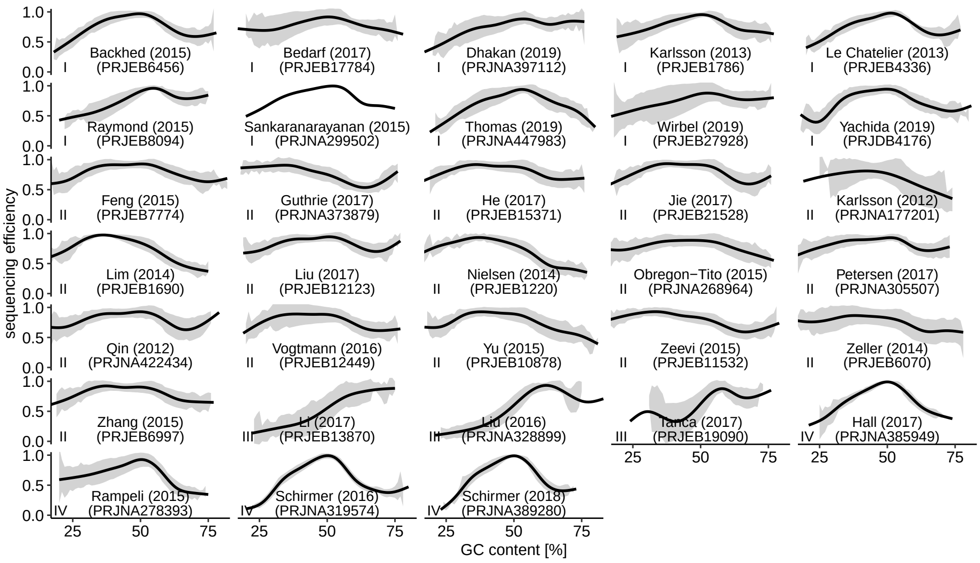

Fig. S3 Individual GC-dependent sequencing efficiencies for the 33 studies shown in Fig. 4. Black lines are averages across the samples in each cluster smoothed with with ggplot's `stat_smooth`, method GAM. The grey areas represent ± one standard deviation around the average.

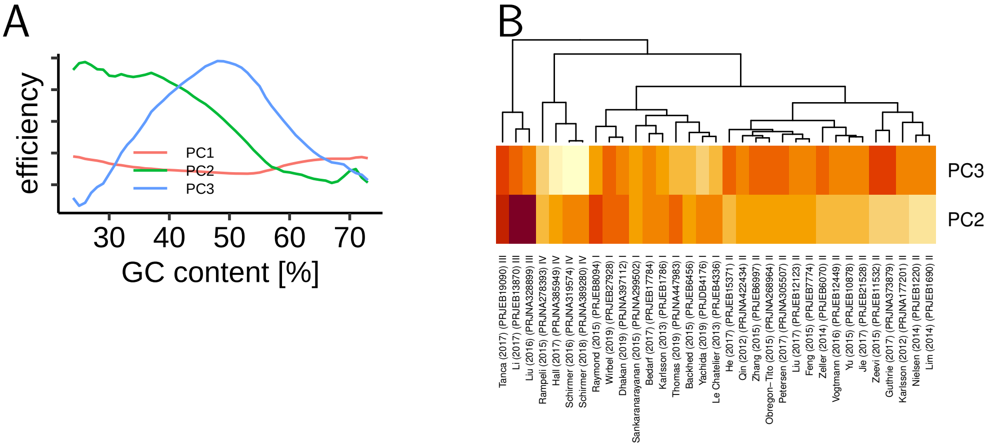

Fig. S4 Clustering of the 33 studies shown in Fig. 4 into 4 clusters. **(A)** First three principal components of the average GC-dependent efficiencies within each study. **(B)** Hierarchical clustering based on the euclidean distances between PC1, PC2, PC3.

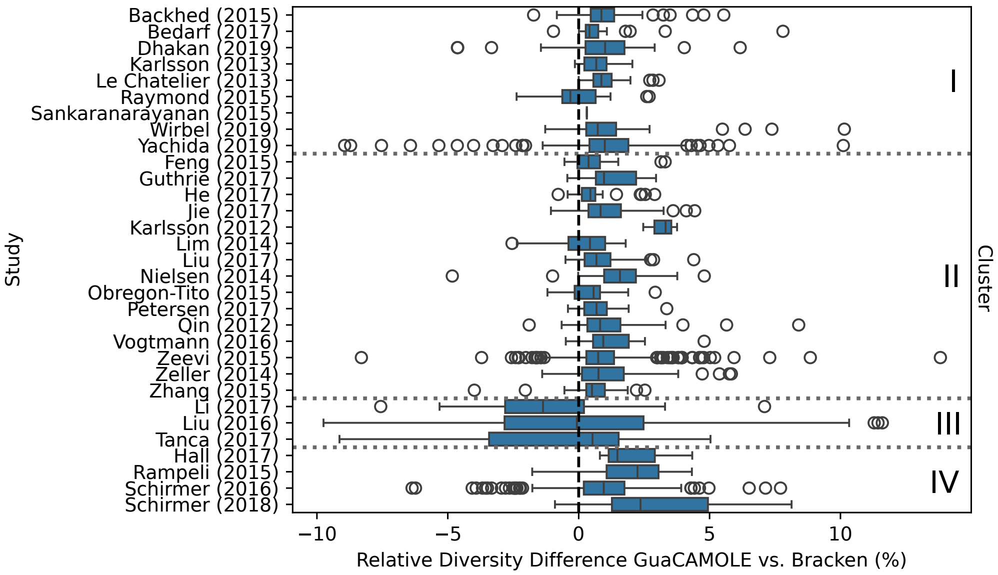

Fig. S5 Difference in alpha diversity across studies Alpha diversities were computed using abundance estimates by GuaCAMOLE and Bracken for each sample. Only samples of healthy individuals were used. Boxplots show the median (center line), 25% and 75% quantiles (hinges) and the furthest point less than 1.5 IQRs (inter-quartile ranges) from the nearest hinge.

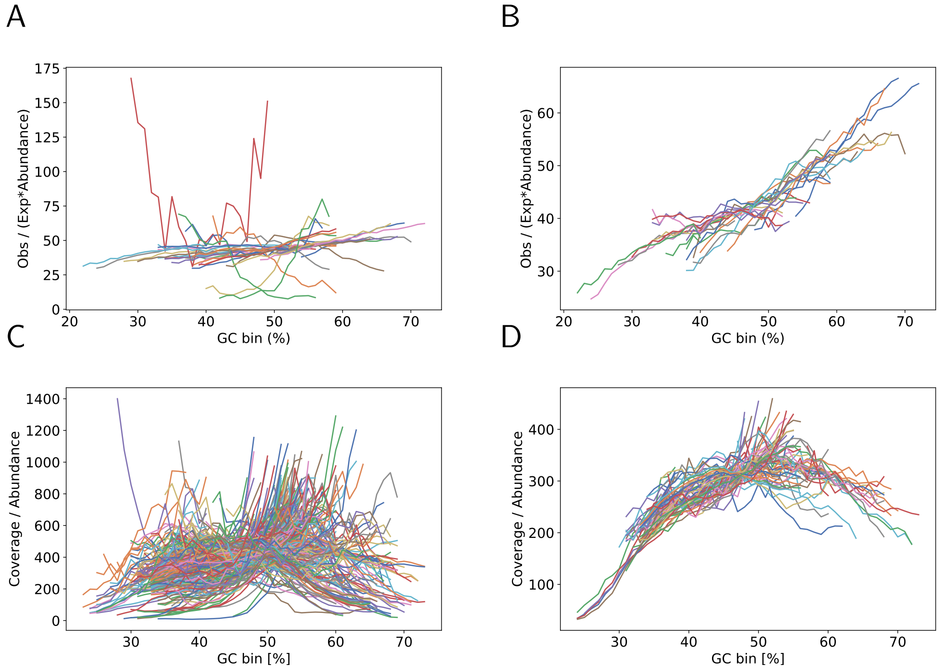

Fig. S6 Inferred taxon-specific efficiencies before and after outlier removal. Y-axis shows η_g as defined in Eq. (7) without the pre-factor C/N. **(A)** Taxon-specific GC-dependent efficiencies for Tourlousse *et al.* protocol DH replicate b before outlier removal. **(B)** Same as (A) after 4 rounds of outlier removal. **(C)** Yachida *et al.* sample DRR127476 before outlier removal. **(D)** Same as (C) after 4 rounds of outlier removal.

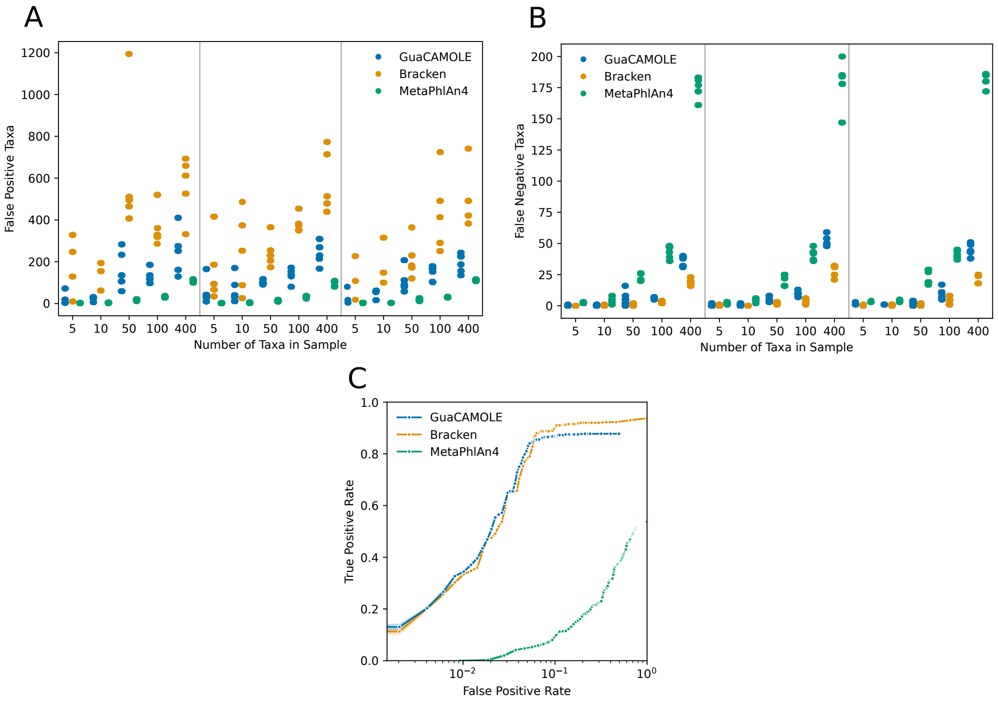

Fig. S7 False positive and negative taxa detection of simulated communities (A) Number of false positive taxa detected by GuaCAMOLE, Bracken (both with minimum read threshold threshold 100) and MetaPhlAn (with default FPKM threshold 1).

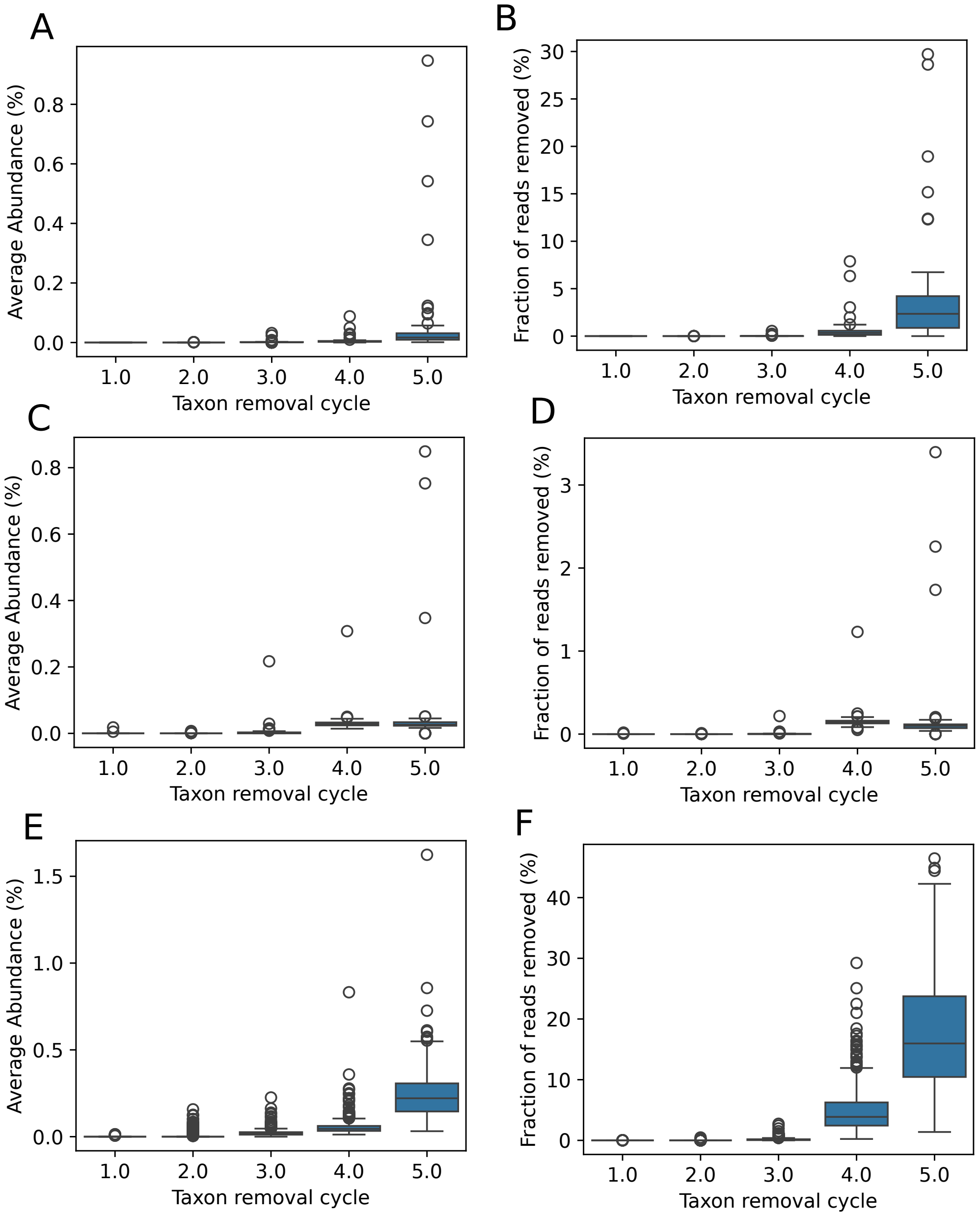

Fig. S8 Average abundance and read fraction removed by each false positive detection cycle. Boxplots show the median (center line), 25% and 75% quantiles (hinges) and the furthest point less than 1.5 IQRs (inter-quartile ranges) from the nearest hinge. **(A)** Average abundance of taxa removed in each cycle by GuaCAMOLE for each sample of the simulated data **(B)** The fraction of total reads removed in each cycle for each cycle of the simulated data (where InSilicoSeq was used) **(C)** Same as (A) but for the [3] data set **(D)** same as (B) but for the [3]. **(E)** Same as (A) but for the [4] CRC data set **(F)** same as (B) but for the [4] CRC data set.

References

[1] Mori, H. *et al.* Assessment of metagenomic workflows using a newly constructed human gut microbiome mock community. *DNA Research* **30**, dsad010 (2023). https://doi.org/10.1093/dnares/dsad010.

[2] Browne, P. D. *et al.* Gc bias affects genomic and metagenomic reconstructions, underrepresenting gc-poor organisms. *GigaScience* **9**, giaa008 (2020).

[3] Tourlousse, D. M. *et al.* Validation and standardization of dna extraction and library construction methods for metagenomics-based human fecal microbiome measurements. *Microbiome* **9**, 1–19 (2021).

[4] Yachida, S. *et al.* Metagenomic and metabolomic analyses reveal distinct stage-specific phenotypes of the gut microbiota in colorectal cancer. *Nature medicine* **25**, 968–976 (2019).
