# Supplementary material for: Genomic GC bias correction improves species abundance estimation from metagenomic data
Source: Nat Commun. 2025 Nov 26;16:10523. doi: 10.1038/s41467-025-65530-4 (PMC12658245; doi:10.1038/s41467-025-65530-4)
Supplement: Supplementary file 2 — Reporting Summary [file 41467_2025_65530_MOESM2_ESM.pdf]

nature portfolio

Corresponding author(s): Florian G. Pflug

Last updated by author(s): Oct 15, 2025

Reporting Summary

Nature Portfolio wishes to improve the reproducibility of the work that we publish. This form provides structure for consistency and transparency in reporting. For further information on Nature Portfolio policies, see our Editorial Policies and the Editorial Policy Checklist.

Statistics

For all statistical analyses, confirm that the following items are present in the figure legend, table legend, main text, or Methods section.

n/a	Confirmed	
☐	☒	The exact sample size (*n*) for each experimental group/condition, given as a discrete number and unit of measurement
☐	☒	A statement on whether measurements were taken from distinct samples or whether the same sample was measured repeatedly
☒	☐	The statistical test(s) used AND whether they are one- or two-sided<br>*Only common tests should be described solely by name; describe more complex techniques in the Methods section.*
☒	☐	A description of all covariates tested
☒	☐	A description of any assumptions or corrections, such as tests of normality and adjustment for multiple comparisons
☐	☒	A full description of the statistical parameters including central tendency (e.g. means) or other basic estimates (e.g. regression coefficient) AND variation (e.g. standard deviation) or associated estimates of uncertainty (e.g. confidence intervals)
☒	☐	For null hypothesis testing, the test statistic (e.g. *F*, *t*, *r*) with confidence intervals, effect sizes, degrees of freedom and *P* value noted<br>*Give P values as exact values whenever suitable.*
☒	☐	For Bayesian analysis, information on the choice of priors and Markov chain Monte Carlo settings
☒	☐	For hierarchical and complex designs, identification of the appropriate level for tests and full reporting of outcomes
☒	☐	Estimates of effect sizes (e.g. Cohen's *d*, Pearson's *r*), indicating how they were calculated

Our web collection on statistics for biologists contains articles on many of the points above.

Software and code

Policy information about availability of computer code

Data collection	Data was downloaded from SRA using sra-tools, otherwise no specific data collection software was used
Data analysis	GuaCAMOLE is available on Github (https://github.com/Cibiv/GuaCAMOLE), the version used for this study has DOI 10.5281/zenodo.17355036. Processed data and scripted required to reproduce all main analyses and figures are available at https://github.com/Cibiv/GenomicGCBiasCorrectionImprovesAbundanceEstimation. Software versions are Python 3.14, numpy 2.3.3, pandas 2.3.3, seaborn 0.13.2, R 4.2.3, ggplot 3.5.2, data.table 1.14.10.

For manuscripts utilizing custom algorithms or software that are central to the research but not yet described in published literature, software must be made available to editors and reviewers. We strongly encourage code deposition in a community repository (e.g. GitHub). See the Nature Portfolio guidelines for submitting code & software for further information.

Data

Policy information about availability of data

All manuscripts must include a data availability statement. This statement should provide the following information, where applicable:

- Accession codes, unique identifiers, or web links for publicly available datasets
- A description of any restrictions on data availability
- For clinical datasets or third party data, please ensure that the statement adheres to our policy

The sequencing data used in this study is publicly available from the short read archive (SRA), the data of Tourlousse et al. (2020) under accession PRJNA650228 and the data of Mori et al. (2023) under accession PRJNA650228. The SRA accessions of all samples used in this study, including the curated colorectal cancer (CRC)

samples from Gupta et al. (2020) and Murovec et al. (2024), together with the processed data and scripts required to reproduce the main analyses and figures of this publication are available at https://github.com/Cibiv/GenomicGCBiasCorrectionImprovesAbundanceEstimation.
www.ncbi.nlm.nih.gov/bioproject/PRJNA650228}{PRJNA650228} and the data of Mori et al. \cite{moriAssessmentMetagenomicWorkflows2023} under accession \href{https://www.ncbi.nlm.nih.gov/bioproject/PRJDB10817}{PRJNA650228}. The SRA accessions of all samples used in this study, including the curated colorectal cancer (CRC) samples from Refs.~\cite{murovecPredictiveModelingColorectal2024,guptaPredictiveIndexHealth2020}, together with the processed data and scripts required to reproduce the main analyses and figures of this publication are available at \url{https://github.com/Cibiv/GenomicGCBiasCorrectionImprovesAbundanceEstimation}.

Research involving human participants, their data, or biological material

Policy information about studies with human participants or human data. See also policy information about sex, gender (identity/presentation), and sexual orientation and race, ethnicity and racism.

Reporting on sex and gender	No new data, in particular no data involving human subjects was generated for this study.
Reporting on race, ethnicity, or other socially relevant groupings	No new data, in particular no data involving human subjects was generated for this study.
Population characteristics	No new data, in particular no data involving human subjects was generated for this study.
Recruitment	No new data, in particular no data involving human subjects was generated for this study.
Ethics oversight	No new data, in particular no data involving human subjects was generated for this study.

Note that full information on the approval of the study protocol must also be provided in the manuscript.

Field-specific reporting

Please select the one below that is the best fit for your research. If you are not sure, read the appropriate sections before making your selection.

[x] Life sciences [ ] Behavioural & social sciences [ ] Ecological, evolutionary & environmental sciences

For a reference copy of the document with all sections, see nature.com/documents/nr-reporting-summary-flat.pdf

Life sciences study design

All studies must disclose on these points even when the disclosure is negative.

Sample size	No new experimental data was generated for this study. The published datasets used were selected to test our algorithm on a wide range of experimental protocols.
Data exclusions	No data was excluded post-facto.
Replication	No new experimental data was generated for this study. The results of our algorithm for existing data are reproducible.
Randomization	No new experimental data was generated for this study, and published datasets were not re-grouped.
Blinding	No new experimental data was generated for this study.

Reporting for specific materials, systems and methods

We require information from authors about some types of materials, experimental systems and methods used in many studies. Here, indicate whether each material, system or method listed is relevant to your study. If you are not sure if a list item applies to your research, read the appropriate section before selecting a response.

Materials & experimental systems

n/a	Involved in the study	
[x]	[ ]	Antibodies
[x]	[ ]	Eukaryotic cell lines
[x]	[ ]	Palaeontology and archaeology
[x]	[ ]	Animals and other organisms
[x]	[ ]	Clinical data
[x]	[ ]	Dual use research of concern
[x]	[ ]	Plants

Methods

n/a	Involved in the study	
[x]	[ ]	ChIP-seq
[x]	[ ]	Flow cytometry
[x]	[ ]	MRI-based neuroimaging

Plants

Seed stocks	Our study does not involve plants.
Novel plant genotypes	Our study does not involve plants.
Authentication	Our study does not involve plants.
